# Repairing chronic myocardial infarction with autologous mesenchymal stem cells engineered tissue in rat promotes angiogenesis and limits ventricular remodeling

**DOI:** 10.1186/1423-0127-19-93

**Published:** 2012-11-12

**Authors:** Pablo Maureira, Pierre-Yves Marie, Fengxu Yu, Sylvain Poussier, Yihua Liu, Frederique Groubatch, Aude Falanga, Nguyen Tran

**Affiliations:** 1 Department of Cardiovascular Surgery, University of Lorraine, Nancy, France; 2 School of Surgery, Faculty of Medicine, University of Lorraine, Nancy, France; 3 INSERM, U961, University of Lorraine, Nancy, France; 4 Department of Nuclear Medicine, Nancyclotep, University of Lorraine, Nancy, France; 5 Service de Chirurgie Cardio-vasculaire, CHU-Nancy, Hôpital de Brabois, Allée du Morvan, Vandœuvre Cedex, 54511, France

**Keywords:** Chronic myocardial infarction, Tissue engineering, Mesenchymal stem cell, Ventriculoplasty

## Abstract

**Background:**

Tissue engineering scaffold constitutes a new strategy of myocardial repair. Here, we studied the contribution of a patch using autologous mesenchymal stem cells (MSCs) seeded on collagen-1 scaffold on the cardiac reconstruction in rat model of chronic myocardial infarction (MI).

**Methods:**

Patches were cultured with controlled MSCs (growth, phenotype and potentiality). Twenty coronary ligated rats with tomoscingraphy (SPECT)-authenticated transmural chronic MI were referred into a control group (n = 10) and a treated group (n = 10) which beneficiated an epicardial MSC-patch engraftment. Contribution of MSC-patch was tested 1-mo after using non-invasive SPECT cardiac imaging, invasive hemodynamic assessment and immunohistochemistry.

**Results:**

3D-collagen environment affected the cell growth but not the cell phenotype and potentiality. MSC-patch integrates well the epicardial side of chronic MI scar. In treated rats, one-month SPECT data have documented an improvement of perfusion in MI segments compared to control (64 ± 4% *vs* 49 ± 3% p = 0.02) and a reduced infarction. Contractile parameter dp/dtmax and dp/dtmin were improved (p & 0.01). Histology showed an increase of ventricular wall thickness (1.75 ± 0.24 *vs* 1.35 ± 0.32 mm, p &0.05) and immunochemistry of the repaired tissue displayed enhanced angiogenesis and myofibroblast-like tissue.

**Conclusion:**

3D-MSC-collagen epicardial patch engraftment contributes to reverse remodeling of chronic MI.

## Background

The reconstruction of a functional myocardium is the ultimate goal of the cardiac tissue engineering. However, the cell/tissue organization of the normal or the failing heart is evolving towards multifaceted changes in hierarchical architecture that leads inevitably to a functional complexity. Today, severe chronic condition of transmural myocardial infarction (MI) is hard to treat with conventional medical/surgical therapies. Some surgical ventriculoplasty techniques as aneurismal zone’s exclusion [1-3], LV parietal resections [4], and/or limited LV dilatation by CorCap device [5] have been proposed to reshape the remodeled left ventricle. Despite some clinical benefits, a wider application of these strategies is somewhat compromised by difficulties inherent to ventriculectomy, condition of the beating heart and large LV scar that might impair thereafter systolic and diastolic functions [6]. Therefore, attempts of cardiac tissue repair are still challenging biotechnological objectives. Parietal tissue re-organization requires innovative solution to enhance the cell component along with (i) reduction in extracellular matrix alteration and with (ii) restoration of metabolic/perfusion conditions in severe infarct areas. Recently developed surgical reconstruction techniques based on the concept of the epicardial “patch treatment” of MI using autologous MSC grafts have been proposed to modify the macroscopic and microscopic parietal constitution of the ventricular defects [7,8]. Several synthetic or biologically derived natural materials including degradable polyesters composed of lactide (PLA) and glycolide (PLG) or collagen have been used as scaffold for stem cells patches [9-11]. Various tissue designs, i.e. sheet, circular ring or complex multi-stack form, etc., have also been tested [10-13]. In preclinical models, this surgical approach of “ventricular bio-assistance” is reported to yield early encouraging results with a low complication rate. However, it should be noted that these studies were performed on the acute MI phase where native cardiac architecture was not yet engaged into deleterious remodeling process. Thus, this promising strategy might particularly be adapted in the case of chronic and transmural myocardial fibrotic scar where the process of regenerative strategies still remains difficult and challenging. In addition, because of uncertainties emanated from first clinical trials with cardiac stem cell therapy [14,15], it has been emphasized that crucial steps toward improving outcomes seen in preclinical studies requires careful establishment of the diagnosis, extent, and severity of MI.

In this experimental study, we have designed a 3D-artificial tissue by seeding autologous MSCs in a collagen network. We hypothesized that surgical engraftment of this patch directly on a chronic and transmural myocardial infarction could positively impact on the left ventricle remodeling. Herein, we used original imaging techniques and bimodal functional assessments to test the feasibility of this strategy and to analyze the relative contribution of those biomaterials on the repair process.

## Methods

### General protocol & animal model

**Figure 1:**
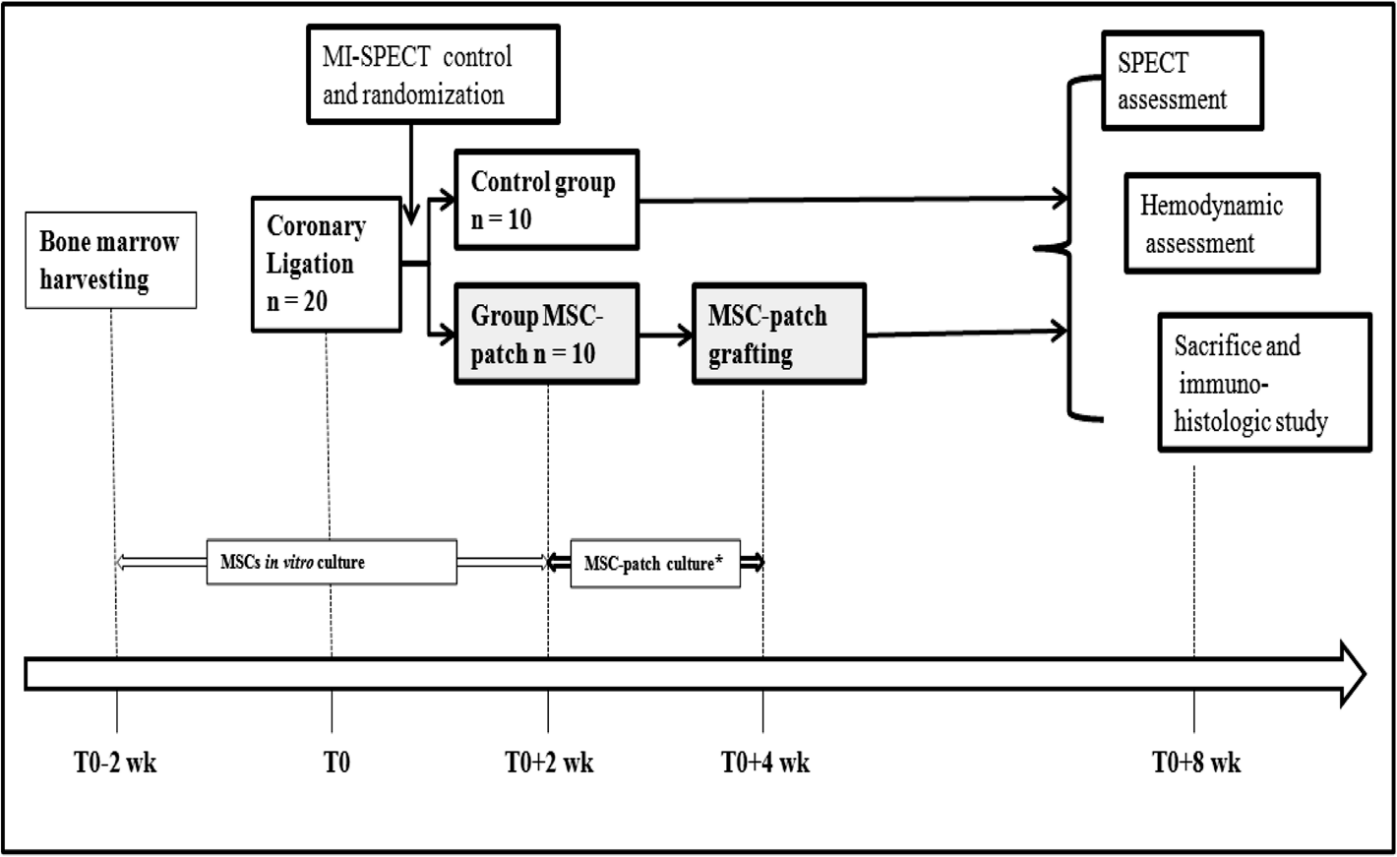
General protocol of the study.

The general protocol is summarized in Figure 1. All experimental procedures were in accordance with our local ethical committee (Number of acceptance: B96-54) and with the regulations of the Animal Welfare Act of the National Institutes of Health Guide for the Care and Use of Laboratory Animals (NIH Publication No. 85-23, Revised 1996). 20 males Wistar rats, weighing 480 ± 15 g at the beginning of the study, were used and coronary ligation was performed for each of them. Briefly, tracheal intubated rats were anaesthetized with isoflurane (3.5%, 1.5 L/min O2) and mechanically ventilated. The heart was exposed through a 1 cm lateral thoracotomy of the fifth inter-rib space. After pericardial incision, the LAD artery was ligated by means of a 7/0 Prolene suture. Four weeks after, all the rats underwent a ^99m^Tc-sestamibi pinhole gated-SPECT according of previously described method [16] to authenticate a transmural chronic myocardial infarction (chronic-MI): the left ventricle segments underperfused (^99m^Tc-sestamibi uptake lower than 50% of the maximum voxel value) where defined as MI segments. Post-MI left ventricular function was assessed by left ventricular end-diastolic volume (LVEDV), left ventricular end systolic volume (LVESV) and left ventricular ejection fraction (LVEF). The rats were then randomly divided into 2 groups: a control group (MI group, n = 10) *versus* a treated group (MI + MSC-patch group, n = 10) in which 3D-MSC-Collagen patch was directly engrafted on MI areas. All the SPECT pre-therapeutic data are depicted in Table 1. 

**Table 1:** **Comparison of quantitative variables extracted from pinhole gated**-**SPECT between MI control rats and MI animals treated with MSC**-**patch one month after patch treatment**: **Data are expressed as mean** ± **SD**

	**MI group** (**n** =**10**)	**MI** + **MSC patch group** (**n** =**10**)	**p**
LV End-diastolic volume (μL)	330 ± 28	295 ± 58	NS
LV End-systolic volume (μL)	179 ± 32	151 ± 43	NS
LV Ejection fraction (%)	47 ± 1	49 ± 7	NS
Number of underperfused MI segments	3.4 ± 1.9	1.8 ± 0.2	0.04
Sestamibi uptake in underperfused MI segments (%)	49 ± 3	64 ± 4	0.02

NS = not statistically significant.

### MSC-patch preparation, characterization and labelling

Two weeks before coronary artery ligation, bone marrow cells were harvested by means of an 18-gauge needle through a perforation of the tibia. After washing with PBS, the cells were transferred into culture dishes containing 10 ml of culture medium (Iscove’s modified Dulbecco’s medium; Gibco Laboratory, Life technologies) containing 10% FBS, 0.1 mmol/L b-mercaptoethanol, 100 U/mL penicillin, and 100 μg/mL streptomycin. Plastic adherent MSCs were cultured up to passage 2 days as previously described [17].

**Figure 2:**
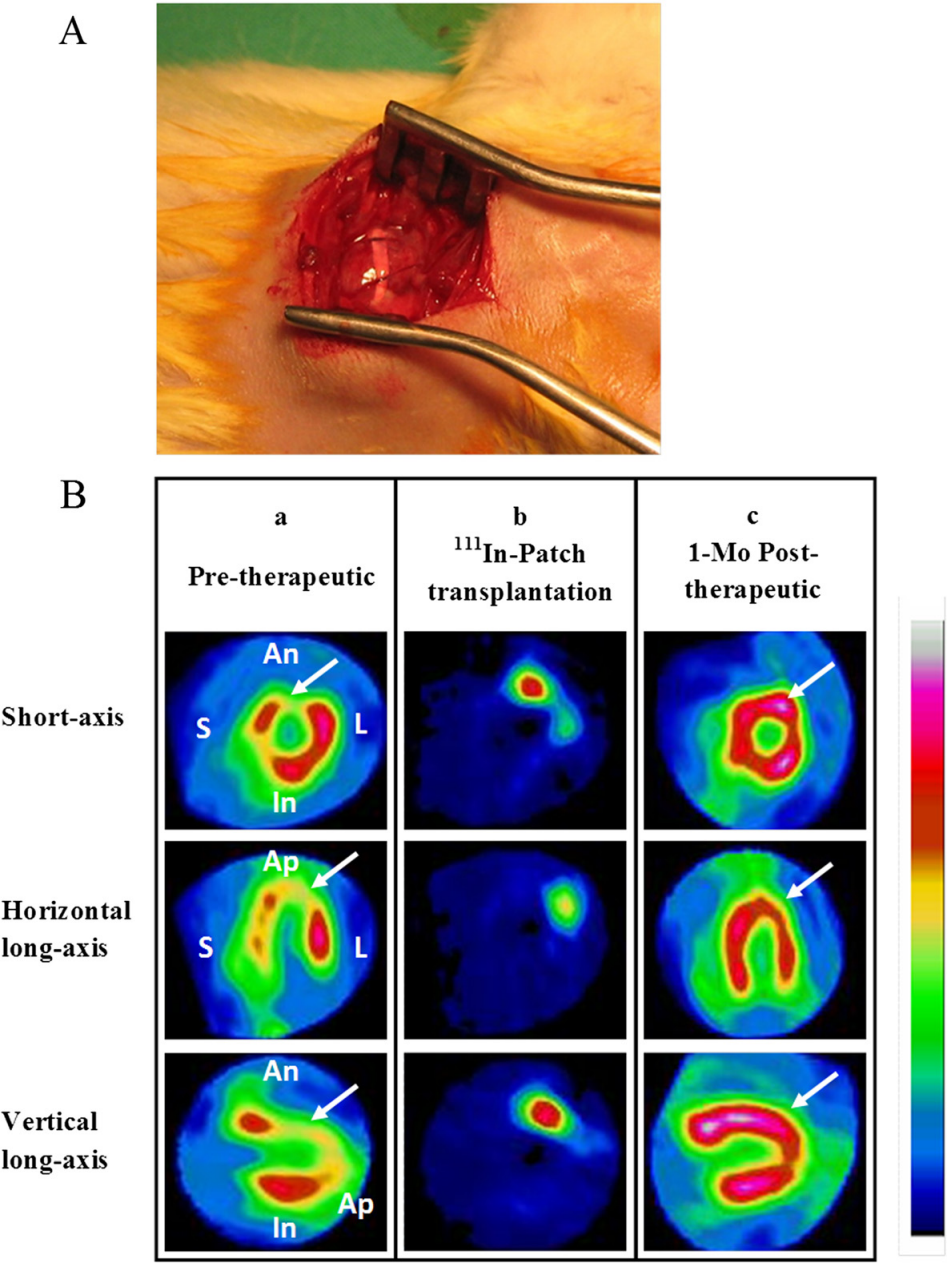
**MSC**-**patch engraftment and SPECT exams. A**: example of ventricular surgical engraftment of MSC-patch. **B**: representative serial images provided by Sestamibi pinhole-SPECT in a treated rat; (**a**) pre-therapeutic perfusion SPECT assessment of chronic MI (MI segments with defect of perfusion, white arrow), (**b**) early post-operative MSC-patch tracking (labelled ^111^In-MSC-patch detected 48-hours after patch engraftment) and (**c**) one month post-operative perfusion SPECT assessment after MSC-patch treatment (MI segments with enhanced perfusion, white arrow).

Thereafter, cells were harvested and 1 million of MSCs were mixed with soluble collagen I solution (3D collagen cell culture system, Chemicon ref: EMC 675). The mixture (150 μL of the ice-cold collagen/cell mix/each mold) was deposed into the casting mold to make a 3-D tissue (MSC-patch, Figure 2). The mixture was allowed to gel at 37°C for 30 min before culture medium was added to the dish. Medium changes were performed after overnight incubation and then every two days. We obtained after 14 day-tissue culture a 4X7 mm rectangular and 1 mm thick patch. Multi-photon confocal microscopy (SP2-AOBS, Leica Microsystems) were used to assess the preservation of some muscle cell markers (desmin and a-smooth muscle actinin). 

In separated experiments, the impact of collagen matrix on changes in the MSC growth, phenotype, and potentiality after 2-Wk tissue culture was also assessed according to our already reported methods [18]. Briefly, for determine the population doubling (PD), cells in P3 were seeded at 5×10^5^ cells per patch and trypsinized after 14 days. Cells were then counted and population doubling time calculated as: t log 2 / log Nf- log Ni with t = time period, Nf = Final cell number; Ni = Initial cell number. To determine de phenotype of MSCs after 14-days seeding on collagen patch, flow cytometry for CD44+, CD90+, CD45- and CD34- (FACScalibur flow cytometer, Becton Dickinson) was used based on the same condition as previously described.

After seeding on collagen scaffold, the potential of MSCs to differentiate into the adipogenic and osteogenic lineages was verified as follows. After 2-wk tissue culture, MSC were enzymatically detached and replated in 60 cm^2^ dishes at different densities and with specialized culture mediums according to the desired differentiation. For adipogenic differentiation, MSCs were seeded at 500 cells/cm^2^ and further cultured for 14 days with standard culture medium. The inception of differentiation was done by complementing standard culture medium with 1 μM dexamethasone, 60 μM indomethacin and 5 μg/ml insulin for 21 days. Cells were then washed with PBS, fixed in 10% formaldehyde, washed with 60% isopropanol and stained with Oil red O Solution (Sigma-Aldrich) to detect lipid droplets within the cells. For osteogenic differentiation, MSCs were seeded at 100 cells/cm^2^ and cultured for 14 days with standard culture medium. Differentiation was induced by supplementing standard culture medium with 60 μM ascorbic acid, 10 mM β-glycerol phosphate and 0.1 μM dexamethasone for 21 days. Cells were washed with PBS and fixed in ice-cold 70% ethanol and stained with Alizarin Red S (pH: 4.1; Sigma-Aldrich) to detect Ca2+ deposits.

### MSC-patch surgical engraftment and SPECT *in vivo* MSCs tracking

Four weeks after coronary artery ligation, rats were anesthetized and a redo thoracotomy was performed under sterile technique. Before patch engraftment, each MSC-patch was labelled with 15 MBq of ^111^In-Oxine (Mallinckrodt Medical B.V.) during a 10 min period as previously described [16]. After exposition of the heart and visualization of the infarcted area, the patch was carefully removed from casting mold and was directly grafted onto the myocardial infarct area following the short axis and attached by two Prolene 7/0 sutures (Figure 2). Animals of MI group underwent the same surgical procedures without patch grafting. Two days after therapy, all treated rats underwent a new pinhole Gated-SPECT using a single-head γ-camera to track labelled MSCs according to our previous published procedures.

### SPECT myocardial assessment and invasive hemodynamic study

One-month after MSC-patch transplantation, *i*.*e*. 2-month after LAD ligation, myocardial perfusion (segments perfusion) and LV function (LVEDV, LVESV and LVEF) were assessed for each rat by ^99m^Tc-sestamibi pinhole gated-SPECT according the same method previously used [16].

One day after SPECT exam, rats have been anesthetized, intubated and mechanically ventilated to allow hemodynamic assessment: the central venous pressure (CVP) was monitored with a catheter advanced in the right atrium through the right femoral vein. Ventricular function was assessed by LVdp/dt max and LVdp/dt min (MP100 acknowledge software, Biopac Systems Inc., Santa Barbara, U.S.) calculated from intra-left ventricular pressure (LVP) measurement using a catheter inserted via the right carotid (sonde 2-F Millar Mikro-Tip®; Millar Instrument Inc., Houston, USA). Measurement of CVP and LVP were conducted at rest and during 15 minutes after an overloading test using administration of sterile NaCl (20 ml/Kg in 1 minute) injected by the left femoral vein.

### Histological and immunohistochemical studies

After hemodynamic assessment, animals were killed by intravenous injection of high pentobarbital and KCl solution. The hearts were harvested, sectioned, were removed and paraffin embedded. To examine the relative contribution of bioengineered patch in MI repair, immunohistochemistry was performed on formalin-fixed, paraffin-embedded tissue sections (5 μm) using the DakoCytomation Envision and Dual Link System-HRP protocol (DakoCytomation, Glostrup, Denmark) in a Dakocytomation AutoStainer (Glostrup, Denmark).

Sections were first deparaffinized and rehydrated. Antigen retrieval was performed by incubating the slides in Tris–citrate buffer pH 6.0 for 20 min at 97°C (PT Link, Dako- cytomation, Glostrup, Denmark). Any endogenous peroxydase activity was quenched by incubating the specimen for 10 min with Dual Endogenous Enzyme Block. Slides were then incubated with desmin (rabbit polyclonal, 1/400, Cell Signaling, Danvers, US) and α-smooth muscle actinin (mouse monoclonal, HHF35, Novocastra, Nanterre, France) for 30 min at room temperature, followed by incubation with the HRP-labeled polymer for 30 min at room temperature. Staining was completed by 10 min incubation with 3, 3-diaminobenzidine (DAB+) substrate-chromogen. Sections were then counterstained with Hematoxylin-Eosin-Safran method. Negative control was made by processing sections in the absence of primary antibody.

### Statistics

The statistical analyses were performed using the program GraphPad Prism v4.03, GraphPad Software Inc. Quantitative variables (perfusion quantity, LVEDV, LVESV, LVEF) were expressed as mean ± SD, and they were compared using non parametric tests: Mann-Whitney tests for unpaired comparisons between treated and non treated animals and Wilcoxon tests for paired comparaisons in each group. For the invasive hemodynamic analysis, a two-way ANOVA was used (the two independents variables were group MI/MI + MSC-patch and basal condition/volume overload test). For each test, a *p* value & 0.05 was considerate as significant.

## Results

### *In vitro* MSC-patch analysis

**Figure 3:**
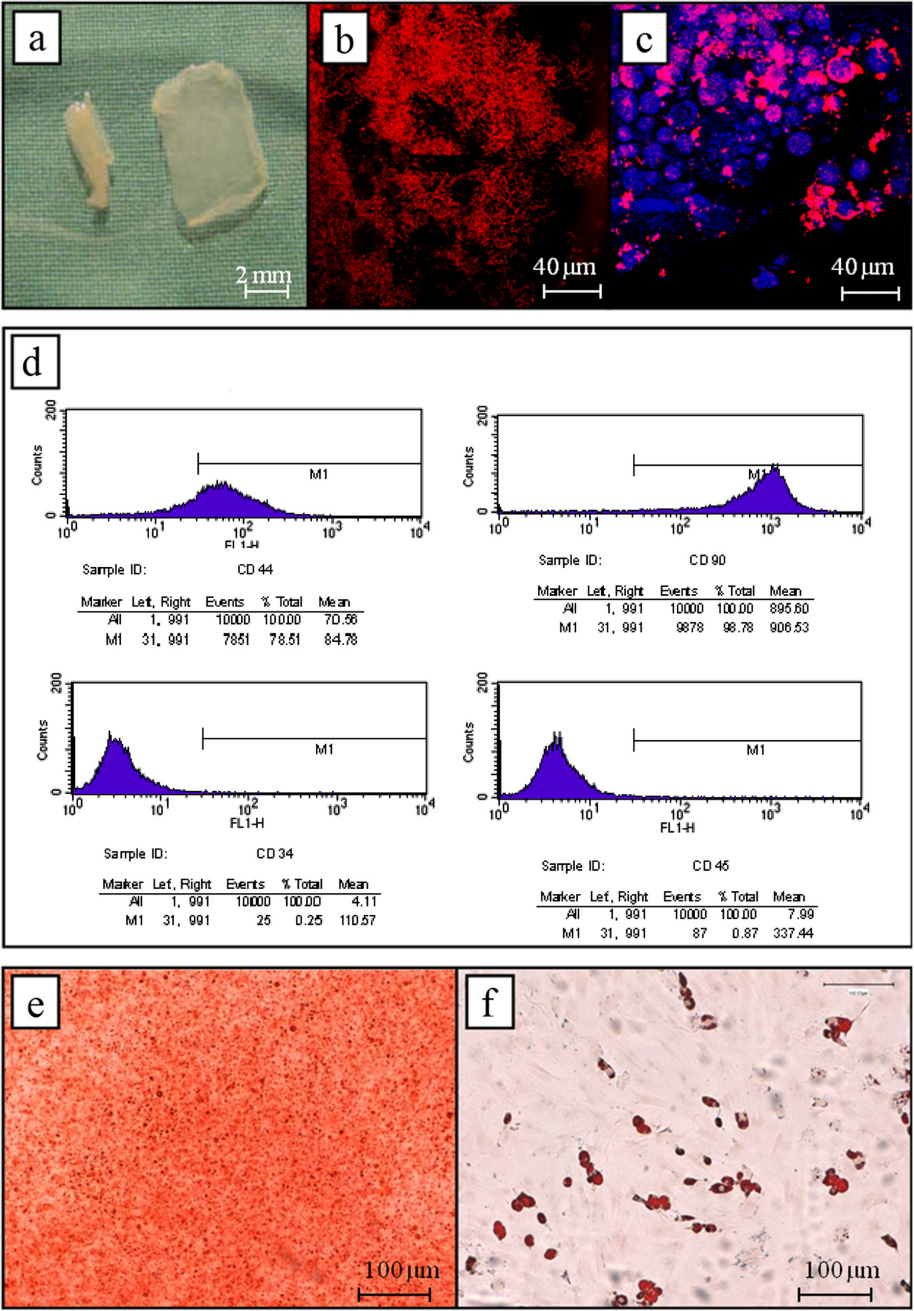
**MSC**-**patch characterization: (a): macroscopic view of 2 weeks *in vitro* cultured 3D**-**MSC patch**, **(b) Collagen structure revealed by near**-**infrared and reflectance confocal microscopy**, **(c) Multiphoton microscopic images of MSCs seeded in collagen patch showing positive staining for α**-**smooth muscle actinin (pink)**; **the nucleus was counterstained by DAPI (blue)**, **(d) FACS analysis of MSC phenotype (CD34**, **CD44**, **CD45**, **CD90) extracted of collagen patch 2 weeks cell culture**, **in (e) osteogenic and (f)**: **adipocyte differentiation potential of MSCs extracted of collagen patch 2 weeks cell culture.**

After 2-wk culturing MSCs on the collagen scaffold, the obtained tissue presented macroscopically as a thin (1 mm rectangular patch (Figure 3a). Near-infrared, reflectance confocal microscopy images (Figure 3b) show the presence of specific collagen matrix fibre that appeared however greatly disorganized and heteromorphic. MSCs presented a retracted and round-shape morphology and still positively expressed some myofibroblastic markers such as a-SM actinin (Figure 3c). FACS analysis has further evidenced a preserved MSC phenotype with various positive (79 ± 1% CD44 and 98 ± 2% CD90, n = 6) and negative (0.25 ± 0.10% CD34 and 0.87 ± 0.05% CD45, n = 6) surface markers (Figure 3d). For examining the growth of MSCs on the collagen patches, the results indicated that the doubling time of MSCs on collagen patches were significantly reduced when compared to conventional 2-D plastic cell culture; the doubling time being 3 time lower (7.9 ± 0.9 days, n = 6) than that observed usually (2.6 ± 0.2 days, n = 6) (t = 5.740, p = 0.0002). In addition, our data seemed indicate that 2-wk plating in collagen did not affect mesenchymal potential since these MSCs showed positive staining for osteogenic and adipogenic differentiation assays (Figure 3e & f). 

### MSC-patch engraftment and post-operative tracking

Two-days after epicardial patch engraftment (see Figure 2A for illustration of surgical procedure), double ^111^In/^99m^Tc-sestamibi pinhole gated SPECT allowed concomitant visualization of myocardial infarction (for example see Figure 2Ba, pretherapeutic panel) and labelled MSC-patch (for example see Figure 2Bb, ^111^In-patch transplantation). These isotopic exams clearly documented a perfect covering of the MI areas by epicardial patch application. The survival rate after two months was 100% in the two groups.

### SPECT data and hemodynamic assessment

**Figure 4:**
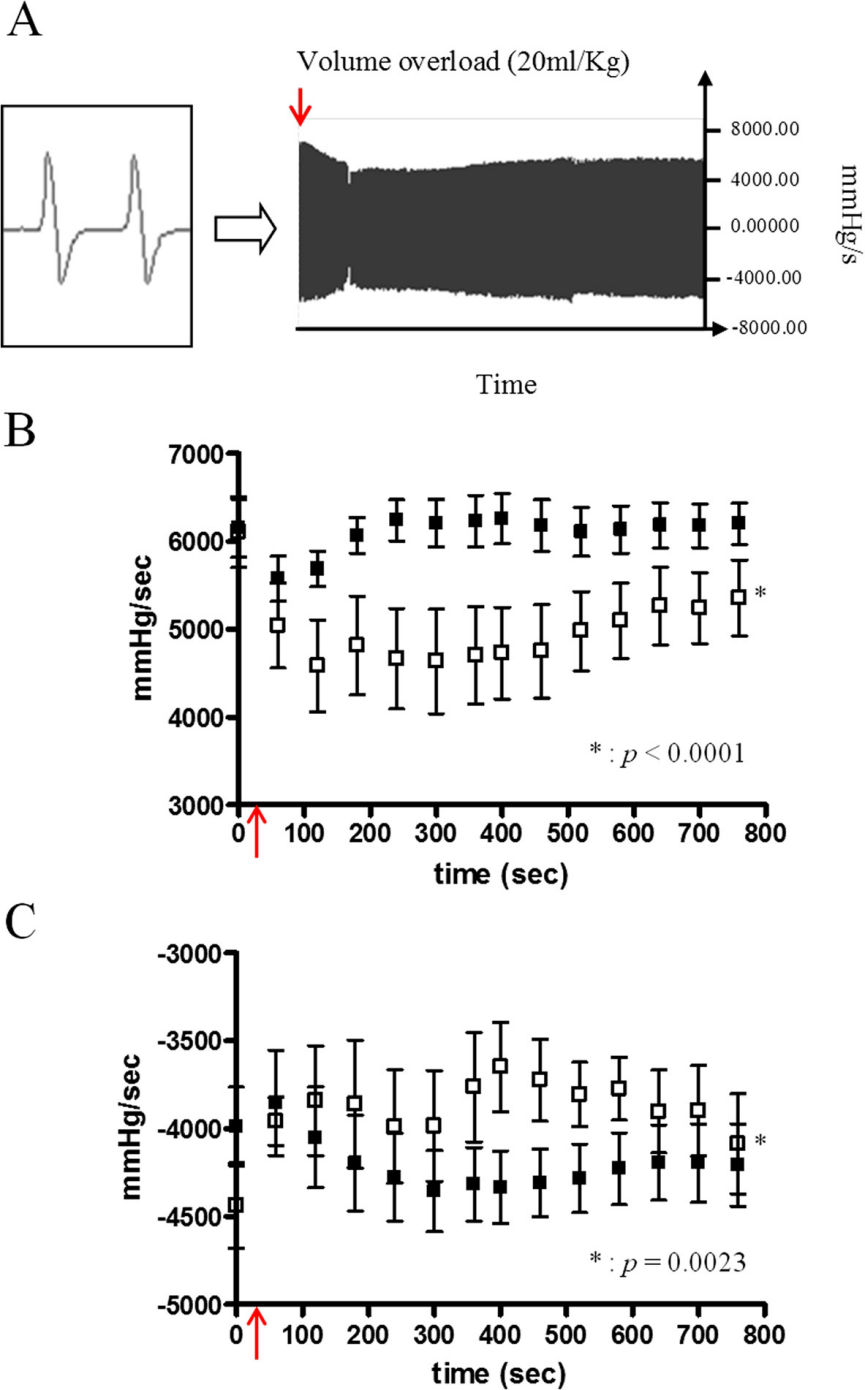
**LV invasive hemodynamic assessment: (A) characteristic example of LVdp/dt measurement, (B) mean LVdp/dt max of MI group (open square) and MSC-patch group (black square) at rest and after the volume overload procedure (red arrow), (C) mean LVdp/dt min of MI group (open square) and MSC-patch group(black square) at rest and after the volume overload procedure (red arrow).** * = P & 0.01 (Two-ways ANOVA).

Figure 2Bc shows an example of SPECT related perfusion improvement in MI segments treated patch seen one month after epicardial engraftment. Comparison of results of MI group and MI + MSC-patch group are resumed in Table 1. Perfusion of infarcted segments was improved in the group treated with MSC-patch while perfusion worsened in the non treated group. One month after treatment, perfusion of MI segments was statistically enhanced for the MI + MSC-patch group (64 ± 4% compared to 49 ± 3% in the control group, p = 0.02). Accordingly, the infarct area was found to be significantly reduced in MI + MSC-patch group (p = 0.04). Similarly, LVEDV and LVESV were smaller in the treated group and LVEF was improved compared to the control group but this was not statistically significant however. The Figure 4 displays hemodynamic measurement at one-month after patch grafting. At rest, basal functional values were not statistically different. Immediately after the volume overload test, preload was enhanced as VCP increase (results not shown). Consequently, left ventricular performance of both groups was altered. However, from the 4^th^ minute after the overload test, LVdp/dt max of treated group was found to return faster to basal value when compared to MI control animals (Two-way ANOVA, F = 53.81, *p* & 0.0001). Similarly, relaxation property (LVdp/dt min) was also found to be significantly improved in the group treated with MSC-patch (Two-way ANOVA, F = 9.61, *p* = 0.0023).

### Histological and immunohistological analysis

**Figure 5:**
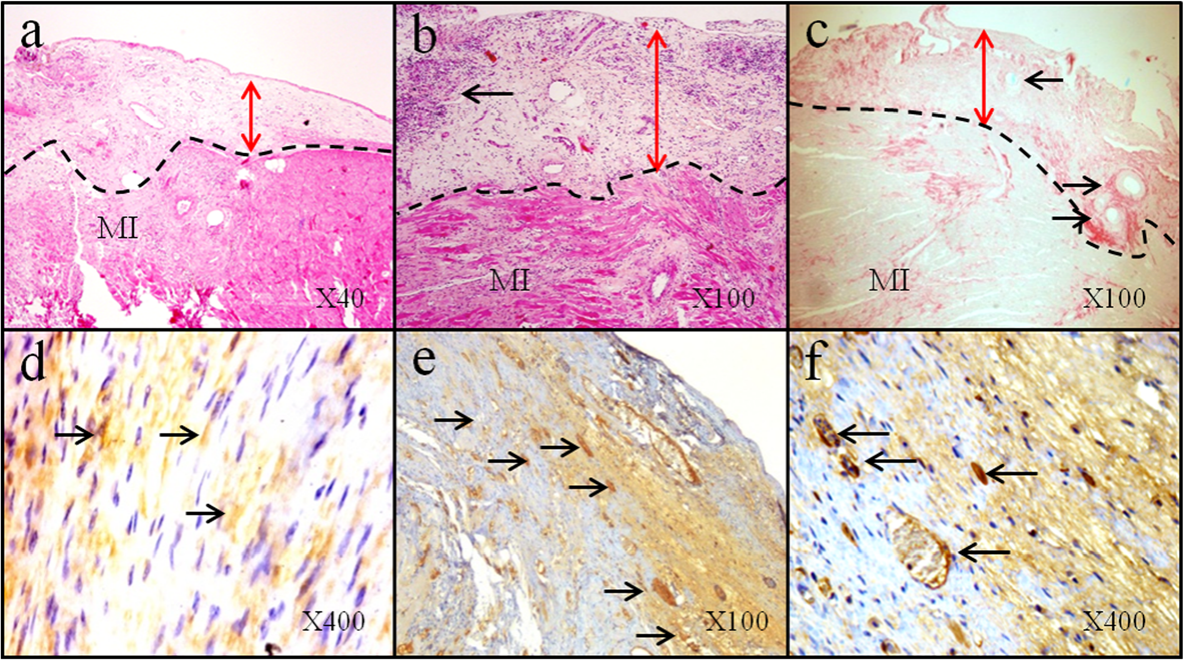
**Histology and immunohistochemical analysis of MI rat heart 1 month after epicardial engraftment of MSC**-**patch:** (**a**) **LV short axis HES assessment showed enhancement of parietal thickness.** The dotted line delimits the engrafted MSC-patch (red arrows) and MI native tissue, (**b**) moderated and localized infiltration with macrophages and polymorphonuclear leukocytes documented in the patch’s area (black arrow), (**c**) Red Sirius assessment showing dense collagen thickness of the engrafted patch and collagen from chronic MI; the black arrows indicate fibrous reactions around 7/0 prolene stitch (blue). Immunohistochemical analysis showed in (**d**) that engrafted MSC’s area expressed desmin (black arrows depict some positive yellow-brownish DAB staining, in (**e**, X100) and (**f**, X400) that engrafted MSC’s area expressed dense tissue positively stained for α-smooth muscle actinin showing dense neo-vessels network (black arrows) between the grafted area and the native myocardial tissue.

Histological examination of transversal sections of the infarcted myocardium treated with MSC-patch showed the good integration of patch and the cell-seeded grafts were adherent to the infarcted area (Figure 5). Tissue remodelling was documented in the sub-epicardial area (Figure 5a, b, and c). There was minor inflammation with infiltration of macrophages in some areas of the patch. The Red Sirius staining clearly authenticated the presence of collagen patch (red colour in Figure 5c) that perfectly covered adjacent native chronic MI. Pathological data have outlined the presence, at the epical targeted graft area, a dense tissue and a heterogeneous architectural reorganization characterized by sheets of cells with elongated nuclei which were strongly positive for some myofibroblastic markers such as desmine and α-smooth muscle actinin, (see Figure 5d, e and f for example). In addition, in sections stained with smooth muscle actinin, the quantification of vessels has demonstrated a marked increase in angiogenesis in territories that beneficiated the bio-assistance with MSC patch compared to MI control group. Values were 7.0 ± 0.9 *versus* 3.2 ± 1.0 vessels number/mm^2^ (t = 9.479, p & 0.0001). 

Planimetry findings further showed an increase in the ventricular wall thickness in cMI group treated by MCS-patch (1.75 ± 0.24 mm *vs* 1.35 ± 0.32 mm, P & 0.05) and a reduction of the left ventricle dilatation; LV diameters being 7.3 ± 0.6 mm in treated group compared to 7.9 ± 0.5 mm seen in control MI group (P & 0.05).

## Discussion

Treating the ischemic heart failure is one of the core targets of new regenerative therapies. However, complexity in ischemic insult has induced various conceptual tools that will inevitably question many of the assumptions of the healing process and the devoted cell or tissue therapeutic strategies [18]. In the recent years, it is urging to better analyze/define the initial status of damaged areas especially in the case of chronic MI. Based on our original small animal SPECT imaging technique, the chronic and transmural MI could be correctly authenticated allowing the central discussion on how the epicardial application of a collagen bio-prosthesis made with MSCs could promote a reverse remodeling process. Outcomes extracted from our multimodal study pointed to a positive impact of MSC-Patch application on the LV reverse remodeling of chronic fibrotic scar including an enhancement of myocardial wall thickness and a reduction of LV dilatation. We also found that these architecture changes were associated with a functional improvement in segmental myocardial perfusion, LV contractility and with tissue reorganization through dense myofibrotic network and angiogenesis.

In the present study, we have adhered to the concept of using collagen in solution as the recipient 3-D structure for seeding stem cells. It is well known now that collagen I is a major component of the cardiac extra-cellular matrix and it provides resistance to deformation [19]. Thus, it porous constitution might facilitate the cell reorganization and the cell exchange of various growth factors and chemokines [20]. Although recent works have suggested that more sophisticated scaffolds forms could be imagined and/or produced with this biomaterial [21], for now we chose a simple, malleable and easily handled forms dictated by the fact that it allowed to avoid ambiguity due to complex structure and to directly address the question that pertains to the very first efficient repair of chronic MI by MSC-collagen patch. In our implementation, when mixing directly MSCs with Collagen I solution, the cell distribution within collagen scaffold was facilitated as evidenced by their homogenous repartition. Physical consistence of the 3D-articicial tissue was particularly dependent of the initial MSC seeded concentration and we found that a 1x10^6^ cells/150 μL of collagen ratio has allowed obtaining a compact and sewable patch. MSCs have been showed to have a 2-wk survival capacity in 3D-collagen [22], which is in agreement with our results. However, such a cell culture in liquid Matrigel affected the MSC morphology (Figure 3b) and cell growth. The spheroid MSC appearance observed can be due to the altered cell-cell attachment caused by unstable collagen 3D architecture (see Figure 3) rather than being a consequence of cell death induction [23]. Although growing slowly (doubling time being 7.9 days compared to 2.6 days in conventional 2D cell culture), these MSCs after plating in collagen still preserved their mesenchymal phenotypes and potentiality.

We tested thereafter the relative contribution of the obtained MSC-patch by using several clinical derived imaging and/or hemodynamic assessments so that relevant decision, regarding the relationship between cardiac function and histopathological changes, could be extracted. After engraftment, our original non-invasive co-localization technique has clearly showed the short-term viable patch within the underperfused and infarcted targeted MI (see Figure 2b for example). The double isotopic detection has already used in our group to successfully follow myocardial engraftment of MSCs alone [16,17]. In this study, this procedure worked well with 3-D tissue and allowed, to our knowledge, the very first in vivo follow-up of engrafted engineered tissue with MSCs thus suggesting the short term survival of these patches within chronic infarcted areas. This is a particularly important issue because the lack of nutrients and oxygen in fibrotic scar might limit direct application of these thick patches. Our in vivo SPECT and immunohistology data have outlined a significant enhancement of myocardial perfusion within the treated areas suggesting that angiogenesis was a preeminent effect in the integration of MSC-patches within chronic MI. Compared with control MI hearts, recipients transplanted with MSC- collagen patch had a higher density of functional microvessels one month after transplantation. We used α-smooth muscle actinin labelling to rather target precapillaries and microvessels networks since it has widely demonstrated that scintigraphic tissue perfusion is strongly correlated with density of functional vessels. It is beyond the scope of this work to identify the specific mechanisms of the short and/or long term inception of angiogenesis, albeit synergic phenomenon resulting from initial surgical inflammatory reaction and angiogenesis due to secretion of cytokines and growth factor (VEGF, FGF.) by MSCs [24,25] might account for the observed local myocardial perfusion. In a recent clinical study using mononuclear cells to treat chronic myocardial infarction, we have further demonstrated that the cell related angiogenesis could be initiated and amplified by an existence of residual metabolic activity [14].

Another observation was that epicardial application of MSC collagen patch has yielded a significant augmentation in the LV thickness suggesting a possible “reverse ventricular remodeling” process after MI. According to pathological analysis, a tissue mesenchymalization as witnessed by dense myofibroblastic tissue was observed at the site of patch application. In addition, although no clear presence of neo-cardiomyocytes was evidenced within engrafted areas, the reshape of the MI defect seemed to have positive impact on reducing the LV expansion and dilatation and on cardiac function as witnessed by our hemodynamic assessments in rats treated with patch tissue. There are no clear consensus concerning the contribution of such reverse remodeling after cell or patch application on LV function [15]. Some authors have suggested that regenerative process of cardiac tissue is underlying the improved function. In a porcine model of hibernating and ischemic heart failure -a tissue condition that is different from chronic transmural infarction in our study- Suzuki et al. [26] have suggested that transplanted MSCs might stimulate proliferation of cardiac progenitors next to the grafted area as well as MSCs differentiation into cardiomyocyte regardless microvascular proliferation. Similarly, Rossini et al. [27] have demonstrated that bone marrow derived MSCs have a better capacity to migrate and might develop *in vivo* a sarcomeric component and have *cardiac stromal cell* morphology. Other works seemed to suggest however that these mechanisms do not occur in sufficiently high frequency to account for the observed functional improvement after MSC administration [28]. There is increasing evidence suggesting that the cardiovascular beneficial effects of stem cell therapy are largely due to the actions of trophic factors and/or paracrine mediators as suggested earlier [24,25]. Moreover, although beneficial impact of patch graft was either on contraction and relaxation function, the fact that the main effect was observed with LVdP/dt max suggested an improvement in the systolic contractile mechanism. However, as discussed above, we have not clearly noticed an organized myocardial tissue formation in the area of patch engraftment. Two hypotheses might be advanced to explain this apparent discrepancy. First, the observed neoangiogenesis might have a beneficial impact on the border areas of MI where hibernating cardiomyocytes could be better rescued by the enhanced myocardial perfusion. Second, changes in physical and morphological properties of passive myocardium such as shear properties and viscoelastic properties might also have a positive impact on the LV overall improvement including systolic function [29]. Indeed, these latter modifications could facilitate the shortening of sarcomeric fibers, reduce the cardiomyocyte’energy consumption and this especially in the case when the preload is augmented after volume expansion. In this study, the patch treated hearts developed a better response to volume overload test suggesting therefore a better myocardial wall compliance that would explain the enhanced V-P relationship.

Our study had however some limits. First, the harvesting time of MSCs before MI inception had no equivalence in clinical process. Second, we did not address the issues of potential arrhythmogenic effect due to the patches made with MSCs. Indeed, heterogeneous electrical conduction might exist within tissue grafting site especially in the area of border zone and could represent a serious risk. Although we did not observed any suspect death after the patch grafting in this study and noticeable arrhythmogenic episodes during 6-mo follow-up in our recent clinical work where MSCs were injected massively in MI areas, we could not rule out this possibility.

## Conclusion

In conclusion, our work support the use of a collagen tissue patch made with MSC as an effective solution to treat chronic MI. We found that MSC-patch can promote reverse remodeling of the infarcted area, probably by directly modifying the architecture parietal but also through paracrine mechanisms, and a functional myocardial improvement mainly through a reverse remodeling process.

## Competing interests

The authors have no competing financial interests.

## Authors’ contributions

PM and NT designed the study, conducted the experiments and wrote the manuscript. PYM provided conceptual suggestions for the study and manuscript preparation. FY, SP, JL, FG and AF participated in the conduction of the experiments. All authors read and approved the final manuscript.
